# Ebola virus disease control in West Africa: an ecological, one health approach

**DOI:** 10.11604/pamj.2015.21.6.6587

**Published:** 2015-05-04

**Authors:** Clement Adebajo Meseko, Adeniyi Olugbenga Egbetade, Shamsudeen Fagbo

**Affiliations:** 1 Regional Center for Animal Influenza and Trans boundary Animal Diseases, National Veterinary Research Institute, Vom, Nigeria; 2 College of Veterinary Medicine, University of Agriculture, Abeokuta, Nigeria; 3 Public Health Veterinarian, Ministry of King Fahad Medical City, Riyadh Saudi Arabia

**Keywords:** Ebola Virus Disease, wildlife, human-animal interface, one health approach, West Africa

## Abstract

The 2013-2015 Ebola Virus Disease outbreak in West Africa had similar nuances with the 1976 outbreaks in Central Africa; both were caused by the Zaire Ebola Virus strain and originated from rural forested communities. The definitive reservoir host of Ebola virus still remains unknown till date. However, from ecological perspective, it is known that the virus first emerged from forest ecotypes interfacing with human activities. As at March 2015, the outbreak has claimed over 9000 lives, which is unprecedented. Though it remains unproved, the primary sources of infection for past and present outbreaks are forest dwelling, human-hunted fauna. Understanding the ecological factors at play in these forest ecotypes where wild fauna interface with human and causing pathogen spill over is important. A broad based One Health approach incorporating these ecological concepts in the control of Ebola Virus Disease can effectively ameliorate or forestall infection now and in the future.

## Commentary

**Figure 1:**
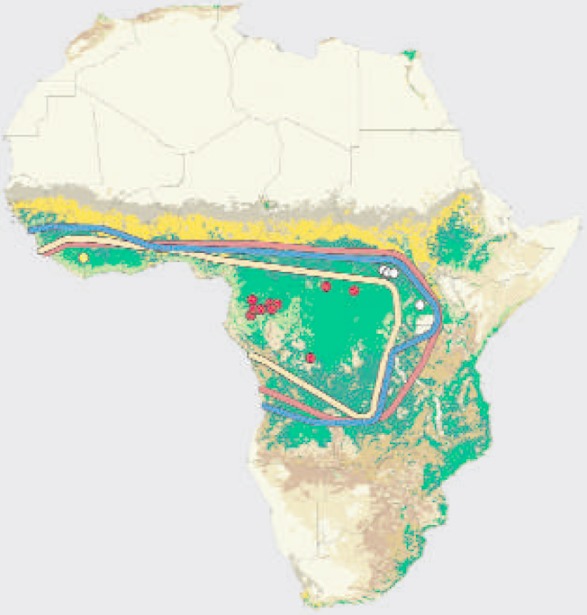
Map of Africa showing West and Central Africa Ebola virus disease region (Leroy et al. 2005. Fruit bats as reservoir of Ebola virus. Nature. 438:575-6)

In 1976, a novel virus that emerged from the Congo basin and was later identified as a new filovirus following virological and immunologic investigation [1]. Initially, details about the virus and the disease it caused were not well understood. However, the 2013-2015 Ebola Virus Disease (EVD) had similar nuances with the 1976 outbreaks as both were caused by Zaire Ebola virus strain (EBOV-Z) and both had similar origin in rural forested communities, where human interface with wild animals is not uncommon [1]. The definitive reservoir host of EVD remain elusive. However, it is ecologically proven to repeatedly emerge from forest ecotypes providing niches for wild fauna. Specifically, EVD derived its name from river Ebola that flows across the forested region of Congo. As at March 2015, the death toll due to EVD in West Africa had surpassed9000 out of over 23,000 infected persons in the worst affected countries of Guinea, Liberia and Sierra Leone [2]. The WHO Director General described the extended 2013 West African outbreak of EVD as unprecedented, and the worst documented in history of mankind and declared the situation as an international health emergency. The episode was summed up as a preventable medical and public health crisis and a social problem [3]. In the midst of tales of woe in West Africa, some countries especially Nigeria where the virus was imported by an air passenger from Liberia seeded an outbreak in Nigeria but this was successfully controlled albeit certain individuals lay down their lives in order that others may live, what in some parlance is referred to as "a good death" [4]. While it was very clear that the virus was imported to Nigeria via air travel, the role of animals was misconceived and negatively communicated to the public: this seriously endangered animal welfare. A better understanding of the ecology of wild animals in relation to pathogen transmission is critical for an overall control of EVD in West Africa. The death-sentence like news of EVD infection fuelled fear and anxiety in many communities in the region. Although in the heat of the epidemic many theories and rumours as regard the causes, consequences and cure of EVD were postulated. Though the epidemic grew and the circulation of rumours around the cause, cure and consequences of intensified, it was clear that EVD transmission was ecologically linked to rural settings where extreme poverty. In such areas, the ability to effectively isolate EVD infected patients and expected infection control capacity were lacking. It was initially difficult to identify animal source(s) of the West African EVD outbreaks. The primary sources of infections were ascribed to wild animals in rural forest where wild animals providing opportunity for zoonotic pathogen emergence. A discerning look at the map of Africa (Figure 1) shows what can be described as the "Ebola forest belt" running across West Africa and Central. Outbreaks of Ebola virus since 1976 to 2015 have been within this forest zone and contiguous areas. Consequently, it can be said that there is something intrinsic about these forest systems that support the sporadic emergence and spillover of EBOV. The forest ecotypes in this zone provide habitats for diverse fauna including primates (such as chimpanzees, gorillas and monkeys) and chiropterids (such as frugivorous and insectivorous bats), Historically, EBOV spill overs and subsequent outbreaks have been associated human interface with these fauna "ensuite" in the forest or its fringes. Bats remain the most favoured putative reservoir host: so far, they are the only specie that can be asymptomatically infected with EBOV. However, it must be reiterated that EBOV infection in bats has only been confirmed via serology and detection of viral RNA; isolation of viable virus remains elusive [5, 6]. As an evidence for the need for a One-Health approach to EVD control, the search for EBOV reservoir hosts has focused not only on forest dwelling mammals but included forest dwelling arthropods and plants [7]. The argument for Ebola as plant virus gets plausible when we consider that intimacy with forest and plant species is a consistent finding in past outbreaks of Ebola and tobacco plant has been successfully used to produce monoclonal antibodies against EBOV which some pharmaceutical companies takes advantage of to produce antibodies to the virus. In the final analysis, the solution to incessant EVD outbreaks in West Africa may also be found in the forest and how animals including man relate with the wilderness.

The biodiversity of the forest ecotype intimately related to EBOV spill-overs and subsequent EVD outbreaks provides habitats to a wide range of fauna and their supporting flora: humans continuously interact with the biotic and abiotic components of this ecotype in complex ways that drive and engender spill-over events. Many of these animals are equally susceptible to zoonotic infectious diseases like Ebola. Hence the combination of susceptibility to agents of infectious disease that can also be transmitted to humans makes wildlife a potentially dangerous reservoir of pathogens. Though many of such wildlife associated zoonotic infections like SIV-HIV, rabies, influenza and corona viruses have been reported to cause morbidity and mortality [8]. However, other than rabies, none precipitate the high case fatality rate known to be associated with the filoviruses EBOV and Marburg virus. With validated therapies or immunoprophylactics lacking and heightened public anxieties, animals, domestic and wild, have been blamed and attacked during the extended West African outbreak. Animal related EVD spillovers are well documented. Subsequent outbreaks have been due to human-to-human transmission starting from 1976. However, it is important to note that not all cases of animal spillovers lead to a full blown outbreak in humans. In 1994, a researcher performing necropsy on a chimpanzee from an observed acute mortality event in forest-dwelling chimpanzees from the Tai forest in Ivory Coast got infected with a novel strain of the EBOV, now known as Taï Forest [9]. In another report in Kikwit, DRC in 1995,EBO-Z strain was responsible for what was then the largest outbreak in Central Africa [2]. The index case of that outbreak was traced to a charcoal maker who had exposures to burrowing species of animals in a forested landscape. Furthermore, another EBOV-Z strain caused fatal disease in Mayibout area of Gabon in 1996, among hunters and family members who butchered and ate a dead Chimpanzee thus triggering chain of invents that led to a large number of human exposure and death. The now extended multinational West African EVD outbreak started off in Meliandou,a rural village in Gueckedou district of Guinea in December 2013 from where it spread to other villages, cities and countries [10]. Though the incidence was not traceable to hunting, carcass contact or eating of primates as in previous cases in West and Central Africa, instead a two-year-old toddler was probably exposed to bat [11]. The child most likely transmitted EVD to sibling, mother and grandmother who all died but not before infecting a village nurse and midwife (health workers) and the transmission chain continued. This event highlights the indispensability of broad based (onehealth) infectious disease cases identification and outcomes in animals and human. From the one health perspective, having a solid understanding of the drivers of spill-over events from forest dwelling fauna to forest interfacing humans is very important if future episodes are to be prevented or controlled. Public health officials need to emphasize that EVD infections result from direct contact with blood, secretion and excretion of infected person or animals or indirectly through contact with environment, objects or fomites that are contaminated. Primary suspects amongst wild animals are mainly primates and bats. Hence EVD infections result from direct contact with blood, secretion and excretion of infected person or animals or indirectly through contact with environment, objects or fomites that are contaminated. In some parts of the world, bats have been hunted as food for a long time where they provide direct source of animal protein in many countries. Unlike human and nonhuman primates, an infected bat may remain healthy. Though evidence of EBOV infection has been documented in various bats species (see Table 1). However, it is yet to be established beyond reasonable doubt that bats are true reservoir of EBOV due to unsuccessful viable virus isolation attempts. This is unlike "a true reservoir" of other infectious diseases such as water fowl that support avian influenza virus without its succumbing to the disease [6]. In the light of this understanding, bats may as well be another incidental host of EVD like primates. Therefore the true reservoir host of EVD may be lying somewhere in the wilderness waiting to be uncovered. Emergence of the EBOV in Guinea highlights the risk of EVD outbreaks in the whole of West African where suspected bats may be found. This risk is not limited to the forest: parks and gardens in West African cities support large colonies of bats. Hence it is not in doubt that an infected bat can readily bite a toddler at fringes of the forest in a rural community. Public enlightenment is necessary to highlight these risks to the populace as they interact with bats and other wild animals.

**Table 1:** Basic ecological and geographical information about bats implicated as Ebola virus reservoirs (adapted from www.batconafrica.net)

Species	Distribution	Habitat	Day roosts	Migratory/non
Epomops franqueti	West and Central Africa mainly	Rainforest, woodlands, and farm bush	Densely foliated large trees	Non migratory
Hypsignathus monstrosus	West and central Africa mainly	Rain forests	Densely foliated large trees	Non migratory
Myonycte ristorquata	Central Africa	Rain forest	In bushes and trees in the forest	Possibly migratory

The one health paradigm provides a platform for public education and control of incessant EVD scourge in West and Central Africa. This entails coming to term with the reality that EVD is first animal virus that is transmitted to human (zoonoses). Globally, zoonoses account for over 60% of all infectious diseases [8] and hundred more are emerging and re-emerging at the human-animal interface. Getting to the root of EVD control therefore requires comprehensive collation/analysis of data that could be used for planning effective intervention at the forest environment, wildlife, animal and human health interface. This is highly pertinent due to the interplay of factors and forces such as climate change, population expansion, and urbanization which exposes mankind to complex varieties of infectious agents in shared environment. The drive for increase food production and ecotourism lures mankind into the forests and natural habitats of many animals. Thus unnatural intermingling at the human-animal interfaces increase chances of zoonotic disease transmission. Many of these infections like rabies, MERS-corona, influenza and Ebola viruses all have their origin in animals. They are driven by ecological, demographic, behavioral and socioeconomic changes. One health approach would prevent devastating morbidity and mortality associated with zoonotic infectious diseases. On the long run, prevention is not only more cost effective, it avoids loss of human life, deprivations and dysfunctional societies. The EVD West African outbreak presents another opportunity to incorporate the One Health paradigm into infectious disease control. Experience from previous outbreaks including lessons about the drivers of disease emergence should be incorporated into public health prevention programs. According to Shakespeare, this must be taken at tide for it to create and achieve desired impact. Specifically, the following need to be considered: sustained homegrown wildlife Surveillance to gather and update data on biodiversity of bats and primates that are found in the region in relation to EBOV activity in animals including human; identification of human-animal interface zones with high propensity for intermingling and consequent exposures to infectious diseases including EVD; community engagement and socioeconomic re-orientation, with a critical analysis of contact frequency and risks between reservoir hosts and susceptible animals or human. In view of the importance of livelihood activities in the emergence of zoonotic pathogen like Ebola, integration of social sciences research particularly focused on human behavior is important. In conclusion, an effective One Health approach should contribute to the prevention of zoonotic disease transmission to the human population. This would avert disastrous health emergencies as witnessed in the West Africa EVD outbreak. To achieve this, human and animal health authorities, environmentalists, community and social group must work together as is enshrined in the One Health concept.

## References

[1] Breman JG, Johnson KM (2014). Ebola then and now. N Engl J Med..

[2] Promed. http://www.promedmail.org.

[3] Chan M (2014). Ebola Virus Disease in West Africa- No Early End to the Outbreak. N Engl J Med..

[4] Mugele J, Priest C (2014). A good Death - Ebola and Sacrifice. N Engl J Med..

[5] Leroy EM, Kumulungui B, Pourrut X (2005). Fruit bats as reservoirs of Ebola virus. Nature..

[6] Olivial KJ, Hayman DTS (2014). Filoviruses in bats: current knowledge and future direction. Viruses..

[7] Swanepoel R, Leman PA, Burt FJ, Zachariades NA, Braack LE, Ksiazek TE, Rollin PE, Zaki SR, Peters CJ (1996). Experimental inoculation of plants and animals with Ebola virus. Emeg Infect Dis..

[8] Jones KE, Patel N, Levy M (2008). Global trends in emerging infectious diseases. Nature..

[9] Formenty P, Hatz C, Le Guenno B, Stoll A, Rogenmoser P, Widmer A (1999). Human infection due to Ebola virus, subtype Côte d'Ivoire: clinical and biologic presentation. J Infect Dis..

[10] Baize S, Pannetier D, Oestereich L (2014). Emergence of Zaire Ebola virus disease in Guinea- Preliminary report. N Engl J Med..

[11] Mari saez A, Weiss S, Nowak K (2014). Investigating the zoonotic origin of the West Africa Ebola epidemic. Embo mol med..

